# Modern Sociology

**Published:** 1903-01-31

**Authors:** 


### MODERN SOCIOLOGY.

### A SLUM EXPERIMENT.

WELL-TO-DO people who hold shares in such societies as the Army and Navy or Civil Service Stores, do not perhaps realise what a blessing, on a smaller scale, such a system of economical buying might be to their poorer neighbours.

Fifty or sixty years ago, a few Lancashire miners realised the advantages of co-operative trading, and started industrial stores. These have now spread all over the country, their members and capital run into millions, and they have been an undoubted boon to thousands of families that would have found it difficult, if not impossible, to save by any other means.

But, so far, the very poor in large towns have been practically untouched by the benefits of co-operation, and it has for some time been the desire of certain progressive men and women connected with the movement, to bring its advantages within the reach of the poorest. With this end in view, careful investigations were recently made by the Women's Co-operative Guild,* and in the course of the inquiry seven towns with large poor areas were visited, and particulars relating to conditions of membership in 96 co-operative societies were collected. The towns chosen were Sheffield, Bury, York, Newcastle, Sunderland, Plymouth, and Bristol. Personal visits to slum-dwellers, interviews with town councillors, school attendance officers, religious and philanthropic workers, and co-operative officials, together with conferences with directors, managers, and employees of stores, formed the basis of the method of inquiry. As a result, some practical recommendations were made, most of which were suggested by methods in use at some co-operative society or other, the chief being: (1) Modifications in the conditions of membership; (2) extension of branch stores in poor neighbourhoods, where low-priced goods in small quantities might be sold; (3) penny banks; (4) clubs.

There were, however, two important suggestions which appeared to many steady-going co-operators as revolutionary and impracticable, and which caused a vast amount of discussion and criticism up and down the country. These were a cooked-meat shop and a co-operative settlement. For some time it seemed as if no society was going to be bold enough to accept and put into practice these ideas, but while others talked, Sunderland society was quietly preparing to act. Five of its 22 branch stores were already in the east-end of the town, and three of these in actual slums. That Sunderland can show as bad slums as one may wish to see is proved by the fact that in 1901 it was the fourth over-crowded town in England—the proportion to the whole number of inhabitants being 30 per cent. In one particular district—Coronation Street—the death-rate is 35 per 1,000 (twice that in the best districts), and measles is four times and consumption and enteric twice as prevalent as over the whole area. One of the branch stores was in this very street, and, notwithstanding the dilapidated and rat-infested condition of the property, it was doing a trade of something like £65 a week. Its success encouraged the members of the society to take a bold step; they determined to put £1,000 into the business, to abandon the old store, buy a plot of land, and put up a large corner block of co-operative buildings. The block was opened in October, 1902. It comprises a grocery shop, a butcher's shop with a cooked-meat department, a hall for meetings, and rooms for two resident ladies. All the chief recommendations of the Guild were thus acted upon, and the block, lighted by electric light, is now the most brilliant spot in Coronation Street and for some distance round. That working-class capital should thus be used to bring the benefits of co-operation to the doors of a class lower down in the social scale, is a departure remarkable enough to attract the notice of all concerned in the problems of poverty, and the experiment will be watched with interest.

Although only started in October, 1902, its success so far has greatly surpassed the brightest dreams of its promoters. The receipts in the grocery store the first week in December were £126 10s. 0½d.; in the butcher's shop, £107 17s. The soup, made from leavings, and therefore nearly all profit, sells like magic, and milk is now being supplied in halfpenny and pennyworths. Possibly coal may be added, to be sold in "bucketfuls." At the same date (December 6th) there were 107 new paid-up members, and 27 more were paying up the shilling entrance fee in threepenny instalments. There were 534 Penny Bank depositors, many of them almost babies, 17 of whom were enrolled in the week for which the figures are given. So great was the furore for saving, when the Bank was opened, that the "Penny Bank Woman," as the children called the lady in charge of this department, attained a degree of popularity hitherto undreamed of.

The settlement quarters, though small, are very prettily decorated, and furnished with co-operatively-made things. The kitchen is coloured pale pink and white, and there are flower boxes in all the windows, which, in spite of noise and smuts, are open day and night. The brilliantly lighted corner is a kind of outdoor club for the neighbourhood; many of the slum dwellers cannot read, but they gather round to spell out the various attractive notices posted in the windows, or to listen to the reading of them, perhaps by a child whose learning is superior to their own.

The "Store Ladies," as the settlers are locally called, entertain their neighbours in the hall, where tea and coffee are drunk out of leadless-glaze cups, and the parties are popularly described as "champion." Classes for girls—singing, cookery, cutting-out, etc.—are held, and the hall is open on Sunday nights for men, when social questions, *e.g.* "Drink," "Housing," "The Unemployed," and other problems of poverty specially affecting east-end dwellers are discussed.

One of the settlers for the first few months is the General Secretary of the Women's Guild, and the other the only lady who sits on the United Board of the Co-operative Union. They find the work one of absorbing interest. Encouragement is taken from the fact that an old house opposite, of a woe-begone appearance, has suddenly started into a coating of Venetian red. The people say "It is because the store has come." The General Secretary writes: "Perhaps other houses will follow suit. Let us hope that repairs will be done inside to damp walls, ceilings that let the water through, and dangerous stairs." Careful statistical records of the houses and people are being kept, and co-operation with local sanitary officials and philanthropic workers forms part of the programme. It need scarcely be added that there is no "charity" in the methods employed.

"To base the foundations of this work on the primary acts of life," writes the General Secretary of the Guild, "gives it a reality that educational work alone can never have. But though we may begin from the sale of a halfpenny black pudding, our idea is that the whole of a poor area should be liberated from all that trades on its ignorance, poverty, and misfortune, and dominated by a co-operative life carried on in the interests of the people themselves."

* Women co-operators are associated for the spread of co-operation and for other social purposes in the Women's Co-operative Guild, which has now 293 branches, with 14,000 members. For information, apply to the General Secretary, Miss Llewelyn Davies, Co-operative Store Coronation Street, Sunderland.

